# Angiogenesis and Proliferation Index in Patients with Acute Leukemia: A Prospective Study

**DOI:** 10.1155/2014/634874

**Published:** 2014-03-31

**Authors:** Prabhavati Jothilingam, Debdatta Basu, Tarun K. Dutta

**Affiliations:** ^1^Department of Pathology, JIPMER, Pondicherry 605006, India; ^2^Department of Medicine, JIPMER, Pondicherry 605006, India

## Abstract

Angiogenesis and proliferation as measured by microvessel density (MVD) and proliferation index (PI) are essential correlates of malignancy. The aim of our study was to evaluate difference between these values in AML and ALL and also to study the modulation in these parameters following achievement of remission in acute lymphoblastic leukemia. Differences between adult and adolescent cases of acute leukemia in relation to these values were also studied. We also tried to assess the relationship between angiogenesis and proliferation. Fifty-five patients with acute leukemia were included in the study. Trephine biopsies were immunostained with CD34 and factor VIIIrAg to demonstrate angiogenesis measured as MVD. Immunostaining with PCNA and Ki-67 was done to study proliferation. We found a significant increase in MVD and PI in cases when compared with controls (*P* < 0.0001). In addition cases with ALL had a significantly higher MVD compared to those with AML (*P* < 0.01). The patients with ALL who went into remission showed a significant reduction in MVD; PI remained high. The cases which did not achieve remission showed no significant reduction in either MVD or PI. All adolescent cases of ALL were similar to adults with respect to MVD and PI.

## 1. Introduction

Angiogenesis is a very important biologic correlate of malignancy whose role is well established in solid tumors and hematological malignancies. There are few studies showing the effect of chemotherapy on angiogenesis in acute leukemia [1]. In spite of extensive studies, the utility of angiogenesis as a prognostic tool in acute leukemia has not been entirely established.

Growth of a tumor as measured by the proliferation index is shown to be an independent prognostic factor in acute leukemia [2]. The relationship between proliferation and angiogenesis has been shown in myeloma [3].

The aim of our undertaking was to evaluate angiogenesis and proliferation in patients with acute leukemia and their modulation at remission in cases with acute lymphoblastic leukemia (ALL). We also studied the relation between angiogenesis and proliferation and if they were individually dependent on the immunophenotypes of acute leukemia. To our best knowledge there are few studies documenting the difference in angiogenesis between acute myeloid and lymphoblastic leukemia [4], as well as therapy related changes in vessel counts and proliferation index in nonpediatric age group patients with ALL. Our study also compares these parameters between adult and adolescent cases of acute lymphoblastic leukemia.

## 2. Materials and Methods

### 2.1. Ethics and Consent

The study was conducted after institutional ethical clearance. Informed consent was obtained from the participants.

### 2.2. Patients and Controls

We did a prospective study over a one-and-half-year period on bone marrow specimens from all patients aged 13 years and above diagnosed with acute leukemia who had adequate bone marrow biopsies. A total of 55 patients fulfilled these criteria and were diagnosed with acute leukemia based on cytochemistry and immunophenotyping (26AML and 29ALL). Cytogenetics and molecular testing was not available in our centre. Nineteen age and sex matched patients who underwent bone marrow study for nonleukemic conditions like pyrexia of unknown origin and staging of neoplasms, with marrow aspiration and biopsy within limits of normalcy, were included as the control population.

### 2.3. Bone Marrow Specimens

Bone marrow aspiration (BMA) and trephine biopsy were obtained from all patients at diagnosis and also from controls. The BMA smears were stained with Leishman and also with cytochemical stains, namely, periodic acid Schiff (PAS), Sudan Black B (SBB), and nonspecific esterase (NSE). The formalin fixed and paraffin embedded bone marrow trephine biopsies obtained from patients and controls were cut into 4 *μ*m thick sections and subjected to haematoxylin and eosin staining, as well as reticulin and immunohistochemical stains.

### 2.4. Immunohistochemistry

Immunohistochemistry (IHC) was done using antibodies directed against CD34 and factor VIIIrAg (fVIIIrAg) as markers for vascular endothelium and PCNA and Ki-67 as markers of proliferation. Immunophenotyping of leukemia was done using a panel of antibodies against TdT, CD34, CD3, CD5, CD8, CD10, CD20, CD117, CD68, fVIIIrAg, and MPO. Streptavidin-biotin method was utilized to perform staining, with DABB being used as the chromogen and haematoxylin as counterstain.

### 2.5. Diagnosis and Classification of Acute Leukemia

Cases with bone marrow blast counts ≥20% were diagnosed as acute leukemia and subtyped after cytochemistry and immunophenotyping according to WHO 2008 classification of tumors of hematopoietic and lymphoid tissues.

### 2.6. Microvessel Density (MVD) Calculation

The CD34 stained sections were examined at 100x magnification for detection of “hot spots,” which are areas with highest vascularization. Four such hotspots were chosen and numbers of microvessels were counted in each of these hotspots at 400x magnification. The MVD was expressed as the average of the total count of vessels. The criteria of a countable microvessel were as follows: (a) any brown stained endothelial cell present individually or in a cluster that was clearly separate from adjacent microvessels, tumor cells, or other connective tissue elements, (b) lack of a muscular wall, (c) presence of a lumina was not a prerequisite, and (d) present away from areas of sclerosis or tumour necrosis [5]. Out of the 55 cases of acute leukemia included in the study, seven showed intense staining of the blasts as well as the background with CD34. In these 7 cases the microvessels were highlighted better with fVIIIrAg staining. The counting of MVD was done using the same method as used for CD34 stained sections. Two observers independently counted the MVD and the average count was taken as the final MVD. There was no significant interobserver variability.

### 2.7. Proliferation Index Calculation

Proliferation index was calculated as the percentage of all cells positive for PCNA, in an area away from necrosis showing maximum intensity. Two hundred cells were counted. In acute leukemia cases as the marrows were packed with blasts the number of normal marrow cells included in PI calculation was insignificant. The average of values of two observers was taken as proliferation index (PI). In one case PCNA immunostaining was suboptimal due to background staining, and hence Ki-67 was used to calculate the PI in a manner similar to that for PCNA.

### 2.8. Post-Remission Marrow Specimens

Out of the 29 patients diagnosed with ALL who underwent chemotherapy using vincristine, L-asparaginase, daunorubicin, prednisolone, and intrathecal methotrexate, bone marrow biopsy following completion of induction phase (day “28”) was available for 14 patients. Eleven of them had adequate biopsy for IHC, six of whom were found to be in complete remission, one case was in CRi as the platelet count, and total count were 71,000/cumm and 1,400/cumm, respectively. These were included in the remission (R) group (*n* = 7). The remaining four cases that did not go into remission were called the nonremission (NR) group (*n* = 4). Trephine biopsies of both the groups were subjected to the same tests used to determine angiogenesis and proliferation. Adult and adolescent cases of ALL were treated similarly. Cases with AML were not treated in our centre and hence postremission marrows were unavailable for assessment.

### 2.9. Statistical Analysis

SPSS and Microsoft Excel were used for analysis with *P* value set at 0.05.

- Individual correlation between Hb, platelet counts, total counts, and blast counts with MVD and also with PI was done using Spearman correlation.
- The significance of difference in MVD and PI, between the groups and subgroups, was assessed using Mann-Whitney test.
- Correlation between MVD and PI was done using Spearman test.
- ALL cases on induction phase, MVD and PI values, at diagnosis and at completion of induction were tested for a significant difference using Wilcoxon matched pairs signed rank test and correlated using Spearman correlation.

## 3. Results

Fifty-five patients with acute leukemia were included in the study. The age/sex characteristics, immunophenotypic distribution, and haematological profile of the acute leukaemia and control groups are given in Table 1.

### 3.1. Leukaemia and Control Groups

- MVD and PI were significantly higher in the entire leukemia population when compared to controls (*P* < 0.001).
- No significant correlation existed between MVD and PI in the leukemia group (*P* > 0.05).

### 3.2. AML, ALL, and Control Group (Figures 1 and 2)

- MVD and PI were found to be significantly higher in cases with AML and ALL when compared individually to their control population (*P* < 0.05). Cases with ALL had a significantly higher MVD compared to AML (*P* = 0.041); however, there was no significant difference in the PI between them (*P* > 0.05).
- Correlation was insignificant between MVD and PI in AML or ALL groups. (*P* > 0.05).

### 3.3. Immunophenotypes of AML and ALL

- The median MVD in patients with T-ALL was higher when compared to cases with B-ALL, but the difference was not statistically significant (*P* > 0.05).
- No significant association was found between the immunophenotype of leukemia and MVD or PI nor was there any correlation between MVD and PI in individual immunophenotypic groups. There was also no significant correlation individually between the haematological parameters and MVD or PI in any group.

### 3.4. Adult and Adolescent Cases with Leukemia

Out of the 55 cases included in the study 11 were adolescents (13–18 years) (7 ALL and 4 AML), and 44 were adults (22 ALL and 22 AML). The MVD and PI were significantly increased in both the groups when compared to their respective controls. No significant difference was observed in the values between adult and adolescent groups, probably indicating that leukemia in adolescents behaves like in the adults.

### 3.5. ALL Cases after Induction Phase Chemotherapy (Figures 3 and 4)

**Figure 3:**
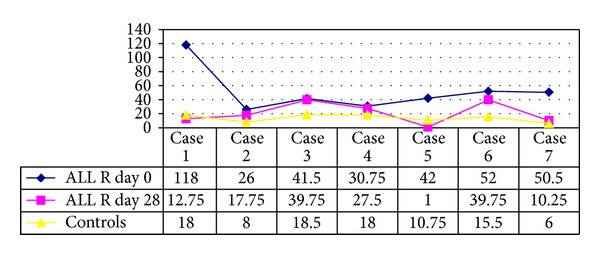
MVD values on day 0 and day 28 in 7 ALL cases showing remission and controls.

**Figure 4:**
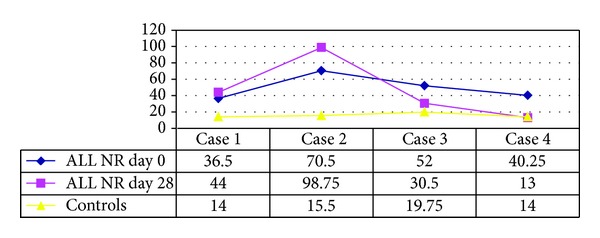
MVD values on day 0 and day 28 in 4 ALL cases not showing remission and controls.

- At diagnosis (day 0) the R group MVD and PI were significantly higher than those of their respective controls. This was contrasting with findings in the NR group which had a significantly higher MVD (*P* < 0.029) and a comparable PI (*P* > 0.05) when compared to their controls.
- There was no significant difference between the MVD or the PI values at diagnosis (day “0”), between the R and NR group.
- The R group had a significant fall in their MVD after induction (day 28) when compared to the day of diagnosis (day 0) (*P* < 0.0156), with values approaching control MVD (*P* > 0.05) (Figure 3). The PI however did not show a significant downtrend (*P* > 0.05), remaining persistently higher than in controls.
- In the NR group, MVD and PI showed no notable decline on day 28 (*P* > 0.05) (Figure 4). Thus MVD was still significantly higher than the controls.
- Vessels in the marrow following remission had wider and empty lumen as they were cleared off blasts and also showed a reduction in endothelial clusters.
- MVD and PI did not show any significant correlation in the R or NR group, neither at diagnosis nor at completion of induction (*P* > 0.05). There was no significant difference in the blast counts or any other hematological parameter between the groups (*P* = 0.49).

### 3.6. AML Cases with Dyspoiesis (Table 2)

- There were six cases of AML with significant dyspoietic changes in the residual marrow hematopoiesis. The MVD and PI in the dyspoietic group were neither significantly different from those of the nondyspoietic group nor from the controls. The nondyspoietic group values were significantly higher than their controls. MVD and PI did not show a correlation in either group.

### 3.7. Control Group

- There was no correlation between the MVD and PI among the controls.

## 4. Discussion

There are a significant number of studies which have shown increase in angiogenesis and/or its mediators in patients with acute leukemia [4, 6, 7]. However, in the present study we have analyzed angiogenesis as well as proliferation, the variation in these parameters between myeloid and lymphoblastic leukemia, and also the alteration in these parameters following chemotherapy in cases with ALL. In addition we have also analyzed the correlation between angiogenesis and proliferation, something that has not been done in published studies. Our study is on nonpediatric cases of acute leukemia, whereas most of the published series on therapy related changes in angiogenesis and proliferation are on childhood acute leukemia. As a part of the project we have studied the behavior of ALL in adolescents with respect to angiogenesis and proliferation index.

A study by Aguayo et al., possibly the only study analyzing angiogenesis between AML and ALL, found no significant difference in the MVD [4]. The current investigation, on the contrary, has shown a significantly higher MVD in cases with ALL compared to AML, a finding not reported so far in the past. We also found that PI did not share this feature. This difference indicates that higher angiogenesis was needed to support ALL and that ALL is more fastidious in its requirement for supporting stromal environment. The higher vascularity could also mean higher drug delivery and hence better chance at remission. We did not find a significant difference in the MVD or PI between any of the immunophenotypic subgroups of AML and ALL in line with other studies [6, 7]. However, our study has limited number of cases in the immunophenotypic subtypes of AML. Angiogenesis has been shown to be related to the degree of anemia, platelet count, blast percentage, and marrow fibrosis [4, 6, 8]. We have not been able to detect any such correlation with any of the hematologic parameters studied.

Studies have tried to elucidate a “mechanical” link, wherein increased marrow cellularity was considered responsible for an increase in angiogenesis, on an increased demand-supply relationship [4, 6, 7]. We found that MVD and PI were not significantly correlated in any of the subgroups and also in the R or NR group, neither at diagnosis nor at completion of induction. Even among the controls, MVD and PI did not show any significant correlation. A study on multiple myeloma found significantly increased MVD in areas with higher Ki-67 values [3]. To the best of our knowledge there have been no studies prior to ours analyzing the correlation between proliferation index and angiogenesis in acute leukemia. VEGF and other angiogenic peptides are involved in autocrine and paracrine stimulation of leukemic cells [7, 9]. Hence we feel a direct relationship between MVD and blast percentage or cellularity might not be possible to establish, as the relation is complex and not linear.

**Figure 5:**
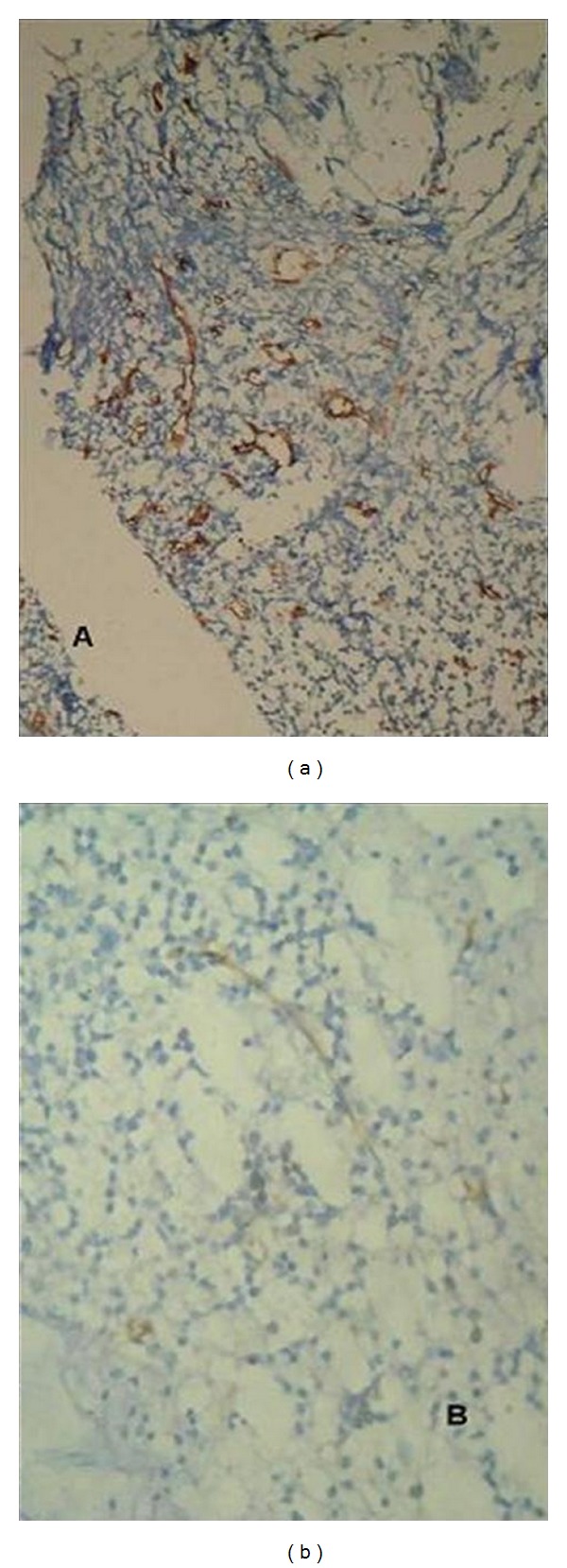
MVD in a case of ALL at diagnosis (a) and reduction following remission (b) (100x). Section stained with CD34 (streptavidin-biotin method).

In our cases of ALL who went into remission, the MVD following induction (day 28) clearly showed a significant downslide when compared to the values at diagnosis (day “0”) (Figure 5) and approached control values. On the other hand, proliferation index continued to be significantly higher than control values as marrow showed accelerated regenerative activity following remission. This finding of a lowered MVD with elevated PI following remission indicates that a lower scale of angiogenesis is required to support high regenerative proliferation, whereas increased angiogenesis is required to support proliferation in leukemia. Our findings are in disagreement with the results of a study by Perez-Atayde et al., who found no significant decrease in the MVD in children with acute leukemia following achievement of remission [10], which again is in contradiction to Pulè et al. who found a significant difference in pediatric age group [11]. The incongruous results could be because of the genetic differences between pediatric versus adult onset ALL and also due to the fact that normal cellularity is significantly different in the two age groups. Ours is probably the first study documenting the changes in MVD in nonpediatric age group cases with ALL following remission. Faderl et al. found that lower levels of VEGF were associated with a poorer prognosis in adults with ALL [12], as against previous studies on childhood ALL which demonstrated poorer prognosis with a higher level of VEGF [13, 14]. Our study shows that leukemia in adolescents behaves similar to that in adults with respect to angiogenesis and proliferation. These studies show that there might be significant difference in angiogenesis in adult and adolescent versus childhood ALL and so might need different approaches with respect to antiangiogenic therapy in these two groups. We have not been able to find a link between angiogenesis and any of the known noncytogenetic prognostic variables in either ALL or AML groups.

In contrast with the R group, the NR group in our study showed no significant reduction (day 28) in either MVD or PI when compared to their values at diagnosis (day “0”). Lack of reduction in MVD was either a consequence or a contributor to the cases not going into remission. Antiangiogenic therapy might be beneficial in such cases if identified at diagnosis.

In the R group MVD and PI were significantly higher at diagnosis (day 0) than their controls. However, in cases which failed to go into remission (NR group), the MVD was significantly above that of controls, but PI was comparable to control values, indicating high vascularity in spite of low proliferative activity, which in turn was probably responsible for failure to achieve remission, as chemotherapeutic drugs aim at the proliferative pool. This could mean, irrespective of proliferative capacity of the blasts, an increase in angiogenesis is needed for their survival. Hence low PI might be useful in identifying cases which might be potential nonresponders to conventional therapy, and in them antiangiogenic therapy might be beneficial as angiogenesis was prominent even on day 28.

Increased angiogenesis has also been found in myelodysplastic syndromes [4, 15] and has been implicated as a poor prognostic marker [15]. In our study the AML cases with dyspoiesis did not have a significantly different MVD or PI when compared to those without dyspoiesis. The dyspoietic group did not have a significantly higher MVD or PI, but the nondyspoietic group showed a significantly higher MVD and PI when compared to their controls. Extrapolating these results to therapeutic domains, in cases with dyspoiesis antiangiogenic therapy might not be beneficial as MVD is not significantly elevated. This needs to be further investigated as our sample size was very limited.

The potential implication of studies on angiogenesis in hematological malignancies lies in its therapeutic application. One of the most critical regulators of angiogenesis is vascular endothelial growth factor (VEGF) which causes endothelial cell proliferation. It is also involved in the “angiogenic loop” responsible for autocrine and paracrine tumor growth and survival [16]. The role of antiangiogenic therapy is based on this interrelationship between tumor and angiogenesis. Angiogenesis is being targeted at two broad levels. One is interfering with VEGF pathway by using antibodies either against VEGF or VEGF receptors (VEGFR) or against tyrosine kinase selective for VEGFR [17]. The second is vascular disruptive agents (VDA), which target nascent or proliferating endothelial cells [18]. These agents are being used in clinical trials especially in cases with AML with favorable results in those refractory to conventional therapy [17, 19].

In summary we have demonstrated a significantly higher angiogenesis in ALL compared to AML, with adolescent cases simulating adults. We have also found a significant drop in MVD following remission, a change not paralleled by proliferation, indicating that normal microenvironment is adept at supporting cells in a regenerating marrow, but not the replicating cells in leukemia. Absence of correlation between MVD and PI points towards a more complex relation between stroma and blasts. If cases could be identified in which snaring the link between them is beneficial, antiangiogenic therapy could increase remission and disease-free rate. Based on the results of our study, ALL cases with low proliferation and AML cases without dyspoiesis could benefit from antiangiogenic therapy. The results however need validation over a large number of cases.

## Figures and Tables

**Figure 1:**
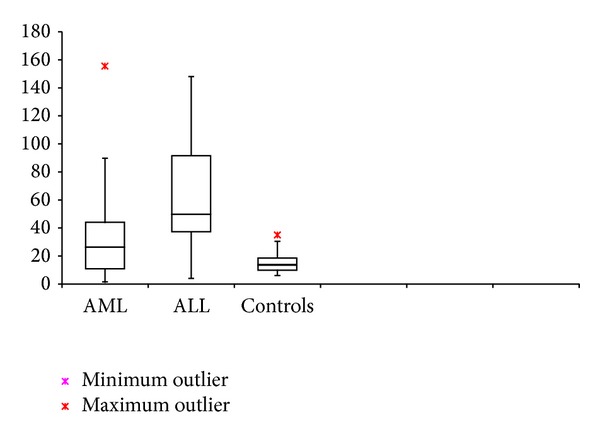
MVD in cases of AML, ALL at diagnosis, and controls.

**Figure 2:**
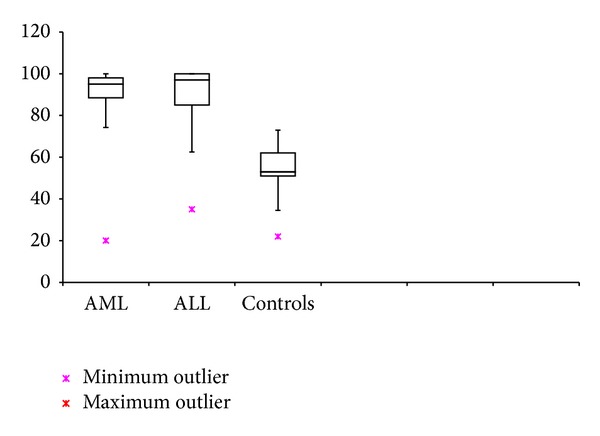
PI in cases of AML, ALL at diagnosis, and controls.

**Table 1:** Patient and control group characteristics.

	Patients with AML (*n* = 26)	Patients with ALL (*n* = 29)	Control group (*n* = 19)
Age in years (median (range))	32 (14–65)	26 (13–66)	32 (13–60)
Sex (males/females)	15/11	18/11	9/10
*Immunophenotypic distribution of acute leukemia cases	4M0, 2M1, 10M2, 3APML, 1M4, 2M5, 2M6, 2M7	T-ALL = 13 B-ALL = 16	Not applicable
Hb g/dL (median (range))	6.5 (3.1–12.9)	6.5 (2.7–13.8)	11 (6.5–14)
TLC per cumm (median (range))	27,150 (900–2.48 lakh)	37,250 (1,200–5.02 lakh)	8000 (6,000–14,000)
Platelet per cumm (median (range))	34,000 (4000–5.21 lakhs)	27,500 (11,000–3.77 lakhs)	1.7 lakhs (1.3–3 lakhs)
Peripheral blood blast % (median (range))	71.5 (1–96)	82.5 (1–97)	NIL
Subleukemic cases % (*n*)	17.86% (5)	16.67% (5)	NA
Bone marrow cellularity	Hypercellular = 25 Myelonecrosis = 1	Hypercellular = 29	Normal for age
Bone marrow aspirate blast % (median (range))	77 (23–96)	91 (52–98)	Nil

M0: AML with minimal differentiation, M1: AML without maturation, M2: AML with maturation, APML: acute promyelocytic leukemia, M4-Acute myelomonocytic leukemia, M5-Acute monoblastic and monocytic leukemia, M6: acute erythroid leukemia, M7: acute megakaryoblastic leukemia, B-ALL: B acute lymphoblastic leukemia, and T-ALL: T acute lymphoblastic leukemia.

**Table 2:** AML cases, dyspoietic and nondyspoietic group, and their controls.

Group	MVD median (range)	*P*	PI median (range)	P
Dyspoietic AML cases (*n* = 6)	42.5 (8.5–155.5)	0.1352	92.5 (20–98)	0.3214
Nondyspoietic AML cases (*n* = 20)	22.125 (1.5–95.5)		96.5 (70–100)	
Dyspoietic group controls (*n* = 6)	13.5 (10.75–19.75)	0.2290	56.5 (52–69)	0.0641
Nondyspoietic group controls (*n* = 13)	13 (6–20.5)	0.0358	56 (22–69)	<0.0001
